# A helical peptide antagonist of the human growth hormone receptor

**DOI:** 10.1210/endocr/bqag022

**Published:** 2026-02-26

**Authors:** Khairun Nahar, Reetobrata Basu, Arshad Ahmad, Joseph A Pettis, Udani Gamage, Justin M Holub, John J Kopchick

**Affiliations:** Department of Chemistry and Biochemistry, Ohio University, Athens, OH 45701, USA; Heritage College of Osteopathic Medicine, Ohio University, Athens, OH 45701, USA; Institute for Molecular Medicine and Aging, Ohio University, Athens, OH 45701, USA; Heritage College of Osteopathic Medicine, Ohio University, Athens, OH 45701, USA; Institute for Molecular Medicine and Aging, Ohio University, Athens, OH 45701, USA; Translational Biomedical Sciences Program, Ohio University, Athens, OH 45701, USA; Department of Chemistry and Biochemistry, Ohio University, Athens, OH 45701, USA; Ohio University Honors Tutorial College, Ohio University, Athens, OH 45701, USA; Department of Chemistry and Biochemistry, Ohio University, Athens, OH 45701, USA; Molecular and Cellular Biology Program, Ohio University, Athens, OH 45701, USA; Department of Chemistry and Biochemistry, Ohio University, Athens, OH 45701, USA; Institute for Molecular Medicine and Aging, Ohio University, Athens, OH 45701, USA; Molecular and Cellular Biology Program, Ohio University, Athens, OH 45701, USA; Heritage College of Osteopathic Medicine, Ohio University, Athens, OH 45701, USA; Institute for Molecular Medicine and Aging, Ohio University, Athens, OH 45701, USA; Molecular and Cellular Biology Program, Ohio University, Athens, OH 45701, USA

**Keywords:** human growth hormone, human growth hormone receptor, site 1-binding helix, peptide antagonist, helical peptide, hydrocarbon stapling

## Abstract

The binding of human growth hormone (hGH) to the human growth hormone receptor (hGHR) is a key endocrinological process that controls critical aspects of cell growth, proliferation, and differentiation. Mechanistically, this sequential, asymmetric binding event involves the interaction between a single hGH molecule and distinct sites (sites 1 and 2) on the extracellular domain of a preformed hGHR homodimer. Our group recently identified S1H, a rationally designed peptide sequence mimetic of the hGH site 1-binding helix (residues 36-51) that disrupts the hGH-hGHR interaction and inhibits hGH-mediated phosphorylation of signal transducer and activator of transcription 5 (STAT5) in hGHR-positive cell lines. Structure–activity relationship studies revealed a positive correlation between helical propensity and inhibitory potency of the S1H peptide, prompting the design of structurally “stabilized” S1H variants (^S^S1H) with improved biological activity. In this study, we employed a chemical strategy, termed hydrocarbon stapling, to generate a series of ^S^S1H peptides that proved to be more helical, proteolytically stable, and biologically active compared to linear (unstructured) S1H. Notably, one ^S^S1H derivative (^S^S1H^B^) inhibited hGH-induced STAT5 phosphorylation in hGHR-positive human bladder cancer cells more effectively than pegvisomant, the only hGHR antagonist currently approved by the FDA. Collectively, our results demonstrate that hydrocarbon stapling improves the antagonistic effects of S1H peptides and elevates their potential as chemical probes to study the molecular mechanisms of hGH signaling. It is also anticipated that ^S^S1H peptides will serve as potent lead compounds for developing next-generation therapeutics designed to treat endocrine disorders that manifest along the hGH-hGHR signaling axis.

The human growth hormone receptor (hGHR) is a homodimeric transmembrane receptor that recognizes and responds to the binding of human growth hormone (hGH), a monomeric serum protein that is released in a pulsatile manner from anterior pituitary somatotrophs (1, 2). High serum levels of circulating hGH can be caused by benign pituitary adenomas and are associated with dysfunctional development in humans. If left untreated, such afflictions often lead to growth disorders (eg, gigantism or acromegaly) and can result in severe physiological complications, including chronic high blood pressure, type 2 diabetes, heart disease, cancer, and premature death (3, 4). Currently, the primary mode of therapy for patients with hypersecreting pituitary adenomas is surgical removal; however, drug treatment is often recommended in cases of unresectable or recurrent tumors. As a consequence, clinical researchers have endeavored to mitigate the functional impact of excess circulating hGH through a variety of inhibitory strategies. These efforts have resulted in the formulation of several drug classes, including somatostatin analogs, anti-hGH antibodies, and hGHR antagonists, that target various facets of the hGH-hGHR signaling pathway (2). For example, somatostatin analogs (eg, paltusotine, lanreotide, and octreotide) are molecules that mimic the structure of somatostatin and function by blocking the release of hGH from pituitary somatotrophs (5-7). Alternatively, anti-hGH antibodies (eg, 13H02) are designed to bind circulating hGH in the bloodstream and neutralize its biological activity by blocking sites required for receptor binding (8). Finally, hGHR antagonists (eg, GF185 and pegvisomant) function by directly targeting the hGHR and competitively block hGH from binding the receptor (9).

The full-length hGHR is a 620-amino acid transmembrane protein comprised of 3 primary domains: an extracellular cytokine receptor homology domain (ECD), a single-pass transmembrane (TM) domain, and a cytoplasmic intracellular domain (ICD) (10). By contrast, hGH is a 191-amino acid globular serum protein comprised of 4 α-helices (HI, HII, HIII, and HIV) that are arranged in a tightly packed antiparallel bundle, and 2 smaller helices that are confined within the so-called “large loop” region (residues 33-75) between helices HI and HII (11). The binding of a single hGH molecule to 2 similar, yet distinct, sites on the homodimeric hGHR-ECD causes the 2 receptor subunits to rotate counterclockwise relative to each other by approximately 45° (1, 12, 13). Previous studies have revealed that the hGHR-ICD interacts with Janus kinase-2 (JAK-2), a protein that exhibits protein tyrosine kinase activity in the cytoplasm (14). The conformational change induced by the binding of hGH to the hGHR activates intracellular JAK-2 proteins complexed with the hGHR-ICD and ultimately results in downstream phosphorylation of signal transducer and activator of transcription 5 (STAT5), a potent transcription factor that facilitates the expression of hGH-responsive genes (15, 16).

The precise mechanisms through which hGH controls its myriad physiological effects are still under active investigation; however, the hGH-hGHR interaction is widely considered to be the principal binding event that triggers intracellular signal transduction cascades associated with hGH-mediated growth and differentiation (12, 17, 18). Accordingly, researchers have focused on developing hGHR antagonists to directly mitigate the biological effects of excess circulating hGH. While the number of *bona fide* hGHR antagonists reported in the literature remains relatively low, 2 major categories of hGHR antagonists have emerged: anti-GHR antibodies and GH analogs. Typically, anti-GHR antibodies are raised against the GHR-ECD and function either by directly competing with GH for binding sites on the GHR-ECD or by preventing conformational changes within the receptor following GH binding. One of the first studies reporting an anti-GHR antibody was published in 1984 with the development of Mab5, a monoclonal antibody that targets an epitope in the dimerization region of subdomain 2 of the GHR-ECD (19). This antibody was developed through hybridoma technology to splenic lymphocytes from BALB/C mice immunized with rabbit liver GHR. While Mab5 was originally intended to be used as an antagonist that blocks GH from binding to the GHR, it has since found use as a reagent for purifying hGH-hGHR complexes (20). Another anti-GHR antibody, anti-GHR_ext-Mab_, targets the dimerization interface between GHR dimers (subdomain 2) and inhibits signal transduction by preventing conformational changes within the receptor following GH binding (21). This conformation-specific inhibitor was found to dramatically reduce GH-induced STAT5 phosphorylation in GH-responsive human fibrosarcoma cells and prevented the formation of GH-induced disulfide linkages within the GHR. More recently, Sun et al reported the discovery of GF185, a monoclonal anti-GHR antibody that targets epitopes localized within subdomain 1 of the GHR-ECD (22). *In vivo* and *in vitro* studies showed that GF185 was able to neutralize GH signaling and inhibit GH-induced proliferation in CHO and Ba/F3 cells.

In addition to anti-GHR antibodies, researchers have also developed synthetic GH analogs that block GH-induced signaling by directly competing for binding sites within the GHR-ECD. The principal example of such an antagonist is Somavert (pegvisomant for injection), a full-length, PEGylated recombinant protein that was developed as the first *bona fide* antagonist of the hGHR (23, 24). Pegvisomant was initially discovered by Kopchick et al in the late 1980s (25), and is currently the only FDA-approved GHR antagonist marketed to treat acromegaly. In terms of structure, pegvisomant is a full-length GH analog that includes 9 amino acid substitutions, including a key G120K mutation that inhibits proper hGHR dimerization (26), and several PEGylations that function to improve biostability. From a therapeutic perspective, pegvisomant has proven useful for attenuating hGH-mediated activation of the hGHR and normalizing circulating IGF-1 in patients with elevated serum levels of hGH (27, 28).

Despite their clinical relevance as hGHR antagonists, monoclonal antibodies and full-length GH analogs are susceptible to many issues that often limit the overall efficacy of protein-based biologics, including costly manufacturing, end-product heterogeneity, immunogenicity, and rapid clearance (28, 29). Therefore, there is significant interest in developing longer-acting GHR antagonists that are not beleaguered by the challenges associated with developing protein-based therapeutics. For these reasons, clinical researchers have turned toward developing peptide-based GHR antagonists that are designed to competitively block the hGH-hGHR interaction (30, 31). One example of such an agent is AZP-3813, a 16-amino acid bicyclic GHR antagonist that binds to the GHR-ECD and prevents endogenous GH from stimulating the production of IGF-1 *in vivo* (32, 33). While the exact sequence of AZP-3813 has not yet been reported, this bicyclic peptide was derived from sequences discovered using a cell-free *in vitro* transcription translation system and was found to bind the hGHR with a *K*_D_ of 1.9 nM. *In vivo* studies showed that maximal suppression of IGF-1 was observed 24 hours after injection of AZP-3813 BID at a concentration of 30 mg/kg, with IGF-1 returning to pretreatment levels 48 hours after drug administration. Notably, there was a maximal 47.2% decrease in IGF-1 levels observed within 24 hours after the first dose of AZP-3813. In contrast, IGF-1 suppression by pegvisomant achieved its maximal inhibitory level (32.5%) 24 hours after its third dose. Recently, a phase 1 clinical trial on AZP-3813 was initiated to evaluate its safety, tolerability, pharmacokinetics, and pharmacodynamics, as well as its potential as an add-on to somatostatin receptor ligand (SRL) therapy.

In addition to their significant therapeutic potential, peptides also hold enormous promise to be developed as chemical tools that can be used to study the molecular nature of protein–protein interactions (34-36). To this end, our group recently developed S1H, a 16-residue linear peptide that is modeled directly from the site 1-binding helix (residues 36-51) of wild-type hGH (30). Notably, the S1H peptide was shown to be effective at inhibiting hGH-mediated STAT5 phosphorylation in various cell lines that express the hGHR and was discovered to be markedly more stable in human serum compared to pegvisomant. Furthermore, the S1H peptide is synthetically tractable, which makes it more cost-effective and processable compared to protein-based biologics (37-39). Structure–activity relationship studies of S1H revealed a strong correlation between helical propensity and antagonist activity, indicating that a helical structure was, in part, required for biological activity (30). To augment these findings, we have now developed a series of “stabilized” S1H derivatives (^S^S1H) that display enhanced helicity, proteolytic resistance, and biological activity compared to linear (unstructured) S1H. These ^S^S1H peptides were generated using a hydrocarbon stapling strategy first reported by Blackwell and Grubbs in the late 1990s (40). One derivative in particular, ^S^S1H^B^, showed greater antagonist activity compared to pegvisomant when inhibiting hGH-mediated STAT5 phosphorylation in an hGHR-positive human bladder cancer cell line (UMUC3). Taken together, these findings suggest that the proteolytic stability and biological efficacy of the S1H peptide can be enhanced through structural preorganization into a helical conformation. Moreover, these results strongly indicate that the site 1 region of the hGHR can be targeted most efficiently with an antagonist that is locked into a helical structure. We anticipate that ^S^S1H peptides can be used not only as chemical tools to study the molecular nature of hGH-hGHR interactions, but also as potent lead compounds in the design of next-generation therapeutics to treat disorders that manifest along the hGH-hGHR signaling axis.

## Materials and methods

The methodologies used in this study are briefly described in the results and figure legends. Antibodies used for western blotting: phospho-STAT5a/b (Y694/Y699) rabbit monoclonal antibody (R&D Systems, Cat# MAB41901, RRID: AB_3658258), total STAT5 (D3N2B) rabbit monoclonal antibody (Cell Signaling Technologies, Cat# 25656, RRID: AB_2798908), and beta-actin (13E5) rabbit monoclonal antibody (Cell Signaling Technologies, Cat# 4970, RRID: AB_2223172). Further details on peptide synthesis, purification, structural characterization, proteolytic stability, and cell treatments are available in the Supplementary Information related to this article. DOI: 10.6084/m9.figshare.31158046.

https://figshare.com/s/63d84854420da4b732ab

## Results

### Hydrocarbon-stapled S1H peptides are derived from linear S1H

**Figure 1:**
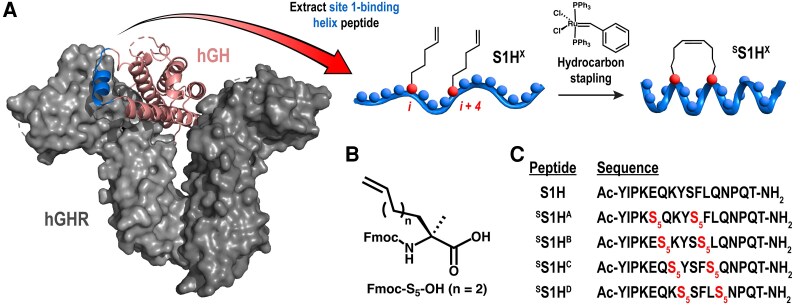
Rational design of stabilized S1H (^S^S1H) peptides. (A) Three-dimensional crystal structure of the hGH (salmon) bound to the hGHR (gray), with the site 1-binding helix (residues 36-51) shown in blue. Image adapted from PDB ID: 1HWG. Extracted S1H peptide sequences were designed with noncanonical S_5_ amino acid substitutions at positions *i* and *i* + 4 within the S1H peptide sequence. (B) α, α-disubstituted noncanonical amino acid (Fmoc-S_5_-OH) used in the synthesis of the ^S^S1H constructs. (C) Sequences of linear and stabilized S1H peptides used in this work.

The linear (unstructured) S1H peptide was originally developed as a direct sequence mimetic of the site 1 binding helix (residues 36-51) of hGH (30). To facilitate hydrocarbon stapling of the S1H peptide, we installed (S)-2-(4-pentenyl) alanine (S_5_), an α, α-disubstituted amino acid functionalized with olefinic sidechains that can be covalently linked through ruthenium-catalyzed olefin metathesis (Fig. 1A). The noncanonical S_5_ amino acid (Fig. 1B) was judiciously placed at positions *i* and *i* + 4 within each peptide sequence so that the hydrocarbon staple would traverse 1 turn of the α-helix following ring closing metathesis (RCM) (40-42). To identify optimal positions for structural stabilization and biological activity, we designed a panel of 4 ^S^S1H variants with the hydrocarbon staples placed at various positions within the S1H sequence (Fig. 1C). Using the three-dimensional crystal structure of hGH bound to the hGHR as a guide (43), we concluded that the region of the S1H peptide with the greatest helical propensity was between residues P38 and P49 (44, 45). Thus, to maximize helical potential upon hydrocarbon stapling, each S_5_ amino acid was positioned between residues P38 and P49 in all ^S^S1H variants. Following the rational design of our peptide library, each construct was synthesized using standard Fmoc-based solid-phase peptide synthesis (46). In order to enhance the stability of our peptides in solution, each peptide was synthesized with an acylated N-terminus and an amidated C-terminus (47, 48) (Supplementary Information (49)). Unexpectedly, the solid-phase synthesis of ^S^S1H derivatives with S_5_ residues at positions K39 and N48 failed (data not shown), and we speculated that this negative outcome was due to the moderately bulky α, α-disubstituted S_5_ amino acid being unable to couple directly adjacent to a proline residue during solid-phase peptide synthesis (45, 50, 51). Once the peptides of desired sequence were successfully synthesized, RCM reactions were performed on solid-phase support using 1st generation Grubbs catalysis (41) (Fig. 1A, Supplementary Information (49)). Following successful completion of the RCM reaction, each peptide was cleaved from the resin and purified to greater than 95% by semipreparatory scale reversed-phase HPLC (Supplementary Information (49), Fig. S1); peptide identities were confirmed during synthesis and purification using MALDI-TOF mass spectrometry (Supplementary Information (49), Table S1).

### Hydrocarbon stapling enhances the helicity of ^S^S1H peptides

**Figure 2:**
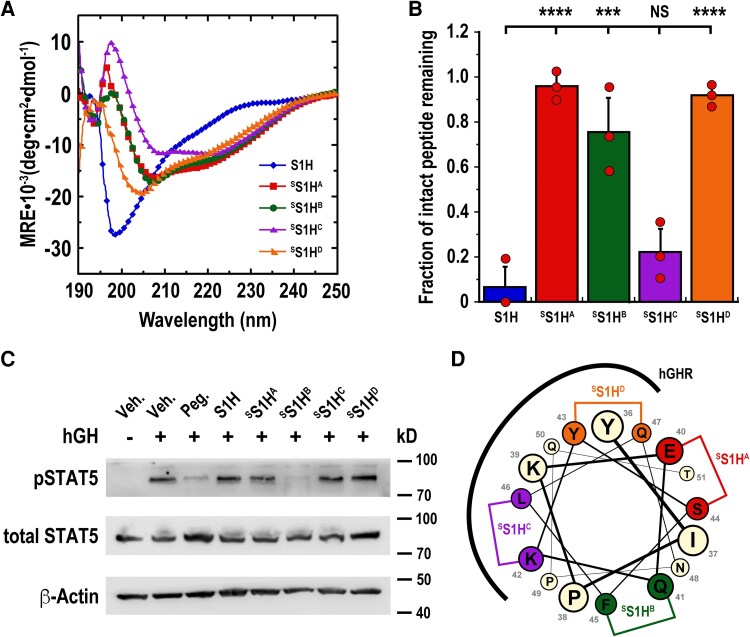
Enhanced helicity, proteolytic stability, and inhibitory effects of ^S^S1H peptides. (A) Circular dichroism spectropolarimetry was used to measure the percentages of S1H variants (40 μM in PBS) maintained in helical conformation. (B) ^S^S1H peptides resist proteolytic degradation by chymotrypsin *in vitro* compared to linear (unstructured) S1H. Bar graph depicts fraction of intact peptide remaining when hydrocarbon-stapled S1H variants were incubated with chymotrypsin. *** *P* < .01; **** *P*  *<* .001; ns: not significant. (C) ^S^S1H peptides attenuate hGH-mediated STAT5 phosphorylation in cultured UMUC3 cells. Serum-starved UMUC3 cells were cotreated with hGH (2.5 nM) and inhibitory peptides (200 nM) for 10 minutes before the intracellular proteins were extracted and quantified by western blot. Veh.: DMSO; Peg.: pegvisomant. (D) Helical wheel representation of the S1H sequence looking down the helix axis from residues Y36 to T51. Position of the hydrocarbon staple is indicated where wild-type residues would be replaced with noncanonical S_5_ amino acids. Assumed ^S^S1H-hGHR interaction interface is based on PDB ID: 1HWG.

Short, linear peptides that contain fewer than 60 amino acids often lack significant structural organization in solution due to the relatively high entropic cost associated with maintaining conformationally-restricted architectures (52). To assess the secondary structures of our ^S^S1H derivatives, we dissolved our peptides in phosphate-buffered saline at a final concentration of 40 µM and generated far-UV circular dichroism (CD) spectra of each variant (Fig. 2A, Supplementary Information (49)). CD spectropolarimetry measurements were plotted as mean residue ellipticity [θ] vs wavelength, and the percentage of ^S^S1H peptide maintained in helical conformations was calculated using Equation 1 (see Supplementary Information (49)). As was observed previously (30), the linear S1H peptide existed predominantly as a random coil in solution, with only 15.7% adopting helical conformations under these conditions. On the other hand, a substantially higher percentage of ^S^S1H peptides were found to be helical under similar conditions. Specifically, it was observed that ^S^S1H^A^ peptides maintained 40.3% helicity, while ^S^S1H^B^, ^S^S1H^C^, and ^S^S1H^D^ peptides each adopted 38.1%, 35.4%, and 32.6% helicity, respectively (Table 1). Importantly, these results indicated that the hydrocarbon staple enhances the percentage of ^S^S1H peptide that adopts helical conformations compared to linear (unstructured) S1H. It should be noted, however, that despite exhibiting an increase in helicity compared to linear (unstructured) S1H, the prolines at positions P38 and P49 likely prevent a higher percentage of ^S^S1H peptides from adopting helical conformations in solution (44).

**Table 1:** Sequence and biophysical data for S1H variants used in this work

Peptide	Sequence	Helical content (%)*^a^*	Fraction of peptide remaining (%)*^b^*	pSTAT5 inhibition (%)*^c^*
S1H	^Ac^YIPKEQKYSFLQNPQT-NH_2_	15.7	6.5 ± 9.2	21.2 ± 6.2
^S^S1H^A^	^Ac^YIPK**S_5_**QKY**S_5_**FLQNPQT-NH_2_	40.3	96.1 ± 5.2	15.0 ± 4.7
^S^S1H^B^	^Ac^YIPKE**S_5_**KYS**S_5_**LQNPQT-NH_2_	38.1	76.0 ± 15.3	46.0 ± 8.4
^S^S1H^C^	^Ac^YIPKEQ**S_5_**YSF**S_5_**QNPQT-NH_2_	35.4	22.3 ± 10.3	12.1 ± 11.4
^S^S1H^D^	^Ac^YIPKEQK**S_5_**SFL**S_5_**NPQT-NH_2_	32.6	91.9 ± 3.9	14.6 ± 4.5

^*a*^Percentage of peptide maintained in helical configuration as measured by CD spectropolarimetry; values extracted from Fig. 2A and calculated using Equation 1 (see Supplementary Information (49)).

^*b*^Fraction of intact peptide remaining following incubation with chymotrypsin ± SD; values extracted from Fig. 2B.

^*c*^Level of pSTAT5 inhibition by S1H peptides in cultured UMUC3 cells ± SD; values extracted from densitometry analysis of Fig. 2C.

### ^S^S1H variants show enhanced proteolytic stability

In addition to enhancing the percentage of ^S^S1H peptides that adopt helical structures in solution, hydrocarbon stapling is also expected to enhance their overall proteolytic stability (53). Therapeutic peptides and protein-based biologics often suffer from limited bioavailability due to protease-mediated degradation and inactivation *in vivo* (54). Generally speaking, proteases require their substrates to be linear (unstructured) in order to make contact and hydrolyze amide bonds (55). In this context, stabilization of peptide secondary structure can effectively shield amide backbones from proteolysis by sequestering its solvent-exposed surface (11, 56). Furthermore, the presence of noncanonical amino acids adjacent to protease cleavage sites has been shown to protect peptides from proteolysis by presenting unfavorable conditions for efficient binding and hydrolysis (57). To assess the proteolytic stability of our ^S^S1H peptides, we first identified potential chymotrypsin and trypsin cleavage sites within the peptides (Supplementary Information (49), Fig. S2). Once such cleavage sites were determined, we incubated our constructs separately in the presence of chymotrypsin or trypsin and compared their degradation products to linear (unstructured) S1H using analytical-scale reversed-phase HPLC (Supplementary Information (49), Figs. S3 and S4). Notably, each ^S^S1H construct was found to be more resistant to proteolysis by chymotrypsin when compared to linear (unstructured) S1H (Fig. 2B). This enhanced resistance was attributed to positioning the noncanonical S_5_ amino acids adjacent to chymotrypsin cleavage sites and a global shielding of the amide backbone by the hydrocarbon staple (Supplementary Information (49), Fig. S2A). In particular, it was observed that only 6.5% of linear (unstructured) S1H remained intact following incubation with chymotrypsin. Alternatively, the ^S^S1H peptides were considerably more stable under these conditions, with 96.1%, 76.0%, and 91.9% of ^S^S1H^A^, ^S^S1H^B^, and ^S^S1H^D^ remaining following incubation with chymotrypsin, respectively (Fig. 2B, Table 1). On the other hand, ^S^S1H^C^ was found to be comparatively less stable in the presence of chymotrypsin, with only 22.3% of the peptide surviving intact following incubation with the enzyme. This is likely due to the potential chymotrypsin cleavage site between amino acids Y43 and S44 not being adjacent to a noncanonical S_5_ amino acid (Supplementary Information (49), Fig. S2A). Contrarily, we found that only one stabilized S1H peptide (^S^S1H^A^) was resistant to trypsin digestion, showing virtually no degradation after incubation with the protease (Supplementary Information (49), Figs. S4 and S5). In this case, the proteolytic stability of ^S^S1H^A^ was ascribed to the trypsin cleavage sites being either adjacent to a noncanonical S_5_ residue or protected by the hydrocarbon staple (Supplementary Information (49), Fig. S2B). Alternatively, peptides ^S^S1H^B^, ^S^S1H^C^, and ^S^S1H^D^ each showed relatively high degrees of degradation following incubation with trypsin that were similar to linear (unstructured) S1H (Supplementary Information (49), Figs. S4 and S5). This instability was likely due to the presence of a potential trypsin cut site (K39) in each stabilized variant that was not adjacent to a noncanonical S_5_ amino acid nor masked by the hydrocarbon linker (Supplementary Information (49), Fig. S2B).

### ^S^S1H peptides inhibit hGH-mediated STAT5 phosphorylation

Once we had generated a series of ^S^S1H peptides that were otherwise more helical and proteolytically stable than linear (unstructured) S1H, we moved to test their biological activity in a cultured hGHR-positive human bladder cancer cell line (UMUC3) (58). Before testing our ^S^S1H peptides as potential hGHR antagonists, we first assessed the cytotoxicity of the constructs by treating cultured UMUC3 cells with varying concentrations of the peptides and monitoring cell viability over 72 hours (Supplemental Information (49), Fig. S6). Here, it was observed that none of the tested ^S^S1H constructs were toxic to cells up to 1 μM, with no significant loss in cell viability at 72 hours. To test the inhibitory potency of our ^S^S1H peptides, cultured UMUC3 cells were cotreated with 2.5 nM hGH and 200 nM of either S1H, ^S^S1H variant or pegvisomant for 10 minutes. Western blotting was then performed in triplicate (*n* = 3) to quantify the level of total and tyrosine-phosphorylated STAT5 (pSTAT5) in treated and untreated cells (Fig. 2C, Table 1, Supplementary Information (49), Fig. S7). Here, we discovered that all peptides effectively attenuated hGH-mediated pSTAT5 levels under these conditions; however, there was substantial variability among the different ^S^S1H variants in the efficacy of hGHR antagonism. For example, linear (unstructured) S1H was found to reduce pSTAT5 levels by 21.2% compared to untreated controls, while ^S^S1H^A^, ^S^S1H^C^, and ^S^S1H^D^ peptides showed more modest antagonism, with 15.0%, 12.1%, and 14.6% reduction of pSTAT5 levels, respectively. We speculate that the diminished activity of these variants stems from the positioning of the hydrocarbon staple, which is located on a face of the helix that is expected to interact directly with the hGHR (Fig. 2D). Notably, it was observed that ^S^S1H^B^ reduced hGH-mediated pSTAT5 levels by 46.0%, which was modestly higher than the inhibitory effect observed with pegvisomant (38.9%) (Supplementary Information (49), Fig. S7). These results strongly indicate that ^S^S1H^B^ acts as an effective hGHR antagonist in cultured human bladder cancer cells and shows significantly greater biological activity than linear (unstructured) S1H.

## Discussion

Despite decades of research, the ability to selectively target and modulate protein–protein interactions that occur across large, shallow interfaces remains a formidable and pressing challenge. Indeed, developing effective modulators of such ephemeral interactions would significantly expand the so-called “druggable” proteome and facilitate the ability to treat innumerable diseases (59-61). From a drug design standpoint, synthetic biologics, antibodies, and recombinant proteins all hold enormous potential to modulate therapeutically-relevant protein–protein interactions (62, 63). Fortunately, the application of such constructs in the clinic and basic research venues is expanding due to the emergence of more sophisticated synthesis protocols and generative computational design strategies (64-66). Although many drug targets previously classified as “undruggable” have benefited from such advancements (67, 68), the number of *bona fide* hGHR antagonists reported in the literature remains frustratingly low. In fact, pegvisomant is the only FDA-approved hGHR antagonist on the market and there is growing interest among clinical researchers to develop next-generation hGHR antagonists that can be used to treat hGH-mediated disease.

Given the limitations that often burden protein-based biologics, peptides represent an attractive alternative due to their synthetic tractability, proteolytic resistance, ability to penetrate cells, and the capacity to mimic the secondary structure of isolated protein interaction domains (54, 69-71). In addition, the modular nature and sequence specificity of peptides allows them to be used as powerful chemical tools to study the molecular nature of protein–protein interactions (34, 35, 72). It is important to consider, however, that the successful development of peptide-based therapeutics presents its own unique challenges, particularly in maintaining structural integrity and enhanced activity in complex biological milieux. Indeed, natural peptide therapeutics often suffer from poor absorption, distribution, and excretion (ADME) profiles, characterized by low oral bioavailability, rapid proteolysis, fast renal clearance, short half-lives, and limited tissue penetration (73). To circumvent these issues, peptide chemists have employed a variety of chemical modification and bioconjugation strategies, including PEGylation (74), installing non-natural amino acids (75), and cyclization (76) to develop peptide-based therapeutics with improved pharmacological properties. It is widely accepted that the pharmacokinetic profile of full-length recombinant proteins can be enhanced due to the presence of PEG groups (77), which are thought to improve overall serum solubility and reduce clearance by protecting exposed amide bonds from proteolysis (74). PEGylation has also been used to enhance the efficacy of therapeutic peptides (78); however, such modifications often have deleterious effects on lower molecular weight compounds, as PEG groups can significantly increase the molecular weight of peptide conjugates and can drastically lower their inhibitory potency (79). As an alternative to PEGylation, cyclization has emerged as an effective strategy to improve the pharmacokinetic properties of peptides through enhanced cell permeability, stabilized secondary structure, reduced proteolysis, suppressed renal clearance, and prolonged half-life (80). Indeed, cyclization introduces conformational constraints that reduce the flexibility and rotational freedom of peptide backbones and can shield exposed amide bonds from proteolysis (81). It has also been demonstrated that cyclization can significantly enhance the pharmacokinetic profiles of therapeutic peptides owing to reduced proteolytic breakdown into smaller molecular weight species (82).

The idea that peptides can be developed as potent hGHR antagonists has been supported by the recent reporting of AZP-3813 (32) and S1H (30). AZP-3813 is a bicyclic peptide that has been shown to target the hGHR with low nM affinity and effectively lowers IGF-1 levels in both mice and humans (32, 33). To the best of our knowledge, however, the sequence and structure of AZP-3813 have not yet been disclosed; it is therefore challenging to define a mechanistic basis for its hGHR antagonism. On the other hand, S1H was developed as a direct sequence mimetic of the site-1 binding helix (residues 36-51) of hGH, making it useful to study the molecular nature of the hGH–hGHR interaction (30). While S1H was shown to lower hGH-mediated pSTAT5 levels in cultured human and mouse cell lines, this construct has proven especially useful as a chemical tool to determine sequence and structural requirements for targeting the site 1 region of the hGHR-ECD. Unfortunately, it is difficult to directly compare the biological effects of AZP-3813 and S1H given the lack of available sequence information and variance in testing parameters. Nevertheless, we acknowledge that each of these constructs holds considerable promise to be developed into next-generation therapeutics that can be used to modulate hGH signaling through the hGHR.

In this study, we pursued a comprehensive investigation to enhance the therapeutic potential of S1H by pre-organizing it into an α-helical structure. Hydrocarbon stapling has been shown to be an effective strategy to stabilize the helical structures of short, linear peptides (42, 56, 83), which can lead to enhanced biological activity when targeting native biomolecular interfaces that involve an α-helix. Given that the structure of the site 1-mini helix (residues 36-51) is predominantly α-helical when situated within the folded full-length hGH protein (43), our strategy focused on stabilizing the secondary structure of S1H by preorganizing the peptide into an α-helix through hydrocarbon stapling. This approach was supported by our previous study demonstrating that the helical propensity of S1H is positively correlated with its biological activity (30). Therefore, we used hydrocarbon stapling to generate a panel of “stabilized” S1H derivatives (^S^S1H) and investigated whether structural preorganization can be used to enhance the helicity, proteolytic stability, and biological activity of S1H.

To accomplish these goals, we extracted the site 1-binding helix of hGH and designed a small library of hydrocarbon-stapled S1H derivatives by introducing olefin-containing amino acids (S_5_) at various positions within the peptide sequence. Following synthesis and purification of the stabilized peptide library, we evaluated their secondary structure in solution using CD spectropolarimetry. All ^S^S1H variants exhibited over a 2-fold enhancement in helical character compared to linear (unstructured) S1H, with a substantial percentage of peptides adopting helical structures in solution. This observation further supports the notion that hydrocarbon stapling increases the proportion of peptides that adopt helical structures and provides a solid foundation for evaluating the proteolytic stability and inhibitory potency of ^S^S1H peptides. Remarkably, all ^S^S1H variants, with the exception of ^S^S1H^C^, showed enhanced resistance to proteolysis by chymotrypsin *in vitro* compared to linear (unstructured) S1H. We reasoned that this enhanced proteolytic resistance resulted from the presence of noncanonical S_5_ amino acids and the hydrocarbon linker shielding susceptible cleavage sites within the peptide sequence. It should also be noted that although only 1 of the ^S^S1H peptides was resistant to trypsin digestion, the overall global enhancement in proteolytic stability indicates that ^S^S1H peptides will likely resist degradation by similar proteases in human serum. It is also notable that ^S^S1H^A^ showed exceptional proteolytic resistance when exposed to chymotrypsin and trypsin. This effect is likely due to the positioning of the noncanonical S_5_ amino acids and hydrocarbon linker within the ^S^S1H^A^ sequence (53). Thus, ^S^S1H^A^ may provide a template for further enhancing the proteolytic stability of other peptide-based hGHR antagonists.

Encouraged by these findings, we moved to assess the biological activity of our stabilized S1H constructs by testing their ability to attenuate hGH-mediated STAT5 phosphorylation in cultured UMUC3 cells. Here, we observed that all stabilized peptides exhibited some inhibitory potential under these conditions, with ^S^S1H^B^ emerging as the most robust hGHR antagonist. Importantly, these effects surpassed the biological activity of linear (unstructured) S1H, indicating that pre-organizing the S1H peptide into an α-helix enhances its overall inhibitory potency. Moreover, the mitigating effect observed with ^S^S1H^B^ exceeded that of pegvisomant, which also showed substantial inhibition of hGH-mediated STAT5 phosphorylation under similar conditions. Owing to the considerable advantages peptides have over recombinant protein-based therapeutics, including ease of synthesis, sequence specificity, and enhanced proteolytic stability (54, 69, 70), we anticipate that ^S^S1H^B^ will serve as a potent lead compound in the search for next-generation hGHR antagonists.

In summary, we have explored hydrocarbon stapling as a strategy to enhance the helical structure, proteolytic stability, and biological activity of S1H, a novel peptide-based antagonist of the hGHR. Our findings demonstrate that hydrocarbon stapling significantly improves the helicity and enhances resistance to proteolysis of the S1H peptide. ^S^S1H peptide variants also exhibited significant improvements as hGHR antagonists without any observable toxicity to cultured cells. Taken together, these findings strongly indicate that enhancing the secondary structure of linear S1H peptides through hydrocarbon stapling can improve their antagonistic effects and biological potency by forcing the S1H peptide to mimic a native protein interaction domain. It is also worth mentioning that although ^S^S1H peptides have been shown to inhibit the downstream effects of hGH in cultured cells, such results may not directly translate to other cell types or *in vivo* systems (84, 85). Therefore, additional studies are required to further characterize how ^S^S1H peptides will affect hGH-mediated processes in other models of endocrine signaling. Accordingly, our lab has initiated extensive studies that compare the biological effects of ^S^S1H peptides in GHR-positive and GHR-negative versions of the same cell line. These studies will allow us to gain better understanding of the mechanistic effects underpinning the molecular basis of antagonism of ^S^S1H peptides. In addition, we are currently testing whether ^S^S1H^B^ will lower serum levels of IGF-1 in mouse models of acromegaly (86). Finally, the promising results observed with AZP-3813 have encouraged us to explore the possibility of using ^S^S1H^B^ as an add-on for SLR therapy or in combination with pegvisomant or AZP-3813 to mitigate the effects of excess circulating GH *in vivo*. We anticipate that the results from these studies will unlock new avenues for developing peptide-based therapeutics that target the hGHR and will facilitate the development of next-generation treatments for hGH-mediated disorders.

## Data Availability

Original data generated and analyzed for this study are included in this article and Supplementary Information (DOI: 10.6084/m9.figshare.31158046; figshare.com/s/63d84854420da4b732ab). Additional data related to this work will be made available upon request to the corresponding authors following publication.

## Antibody research resource identifiers

All antibodies used in the reported research have been identified with Antibody Research Resource Identifiers and appear in the Materials and Methods and Supplementary Information (DOI: 10.6084/m9.figshare.31158046; figshare.com/s/63d84854420da4b732ab) of this manuscript.
